# Editorial: Role of extracellular vesicles in inflammation

**DOI:** 10.3389/fimmu.2026.1825422

**Published:** 2026-03-23

**Authors:** Dhanu Gupta, Bruno D’Agostino, Camilla Margaroli

**Affiliations:** 1 Department of Laboratory Medicine, Karolinska Institutet, Stockholm, Sweden; 2 Department of Paediatrics, University Of Oxford, Oxford, United Kingdom; 3 Department of Environmental, Biological and Pharmaceutical Sciences and Technologies, University of Campania Luigi Vanvitelli, Caserta, Italy; 4 Department of Pathology, University of Alabama at Birmingham, Birmingham, AL, United States

**Keywords:** extracellular vesicles (EVs), host-pathogen interaction, inflammation, lung diseases, mesenchymal stem cells, outer member vesicles (OMV), wound healing

Inflammation remains one of the most fundamental biological processes of homeostatic health and a pathological driver of many diseases. Traditionally defined as the body’s coordinated response to injury or infection, inflammation is a complex cascade of cellular signals, molecular messengers, immune activations, and tissue responses. Over the past decade, there has been a paradigm shift in which intercellular communication in inflammation, including host-pathogen interactions, is not driven solely by cytokines, chemokines, and cell-surface interactions, but also by nano-sized biological particles known as extracellular vesicles (EVs). This Frontiers Research Topic entitled *“Role of Extracellular Vesicles in Inflammation”* provides an overview of cutting-edge research spanning from the role of microbial EVs in modulating inflammatory responses to potential therapeutic approaches using stem cell-derived EVs.

One of the central themes emerging in this Research Topic is how EVs actively regulate both acute and chronic inflammation. For example, Bateman et al. have shown that EVs shed from bronchial epithelial cells promote inflammasome activation in chronic obstructive pulmonary disease (COPD), while Rasmussen et al. have shown that exposure to e-cigarette vapor exacerbates the release of proteolytic EVs by airway neutrophils.

In other contexts, EVs can also propagate inflammation induced by pathogens.Hirsch et al. uncovered that EVs released by cystic fibrosis airway epithelial cells upon infection with *Pseudomonas aeruginosa* trigger neutrophil activation and downstream inflammatory cascades. This suggests that beyond serving as markers of disease, EVs can be active participants in perpetuating inflammation.

Inflammatory responses are fundamentally about cellular crosstalk, and EVs act as currency in this dialogue. Immune cells such as macrophages and mesenchymal stem cells (MSCs) release EVs that carry specific microRNAs and proteins capable of reprogramming recipient cells (Yang et al.). For example, Zhou et al. showed that MSC-derived EVs can deliver regulatory microRNAs to immune cells that suppress inflammatory gene expression and promote resolution. This is especially true in contexts like wound healing and autoimmune inflammation, where a controlled dampening of the immune response is essential for tissue repair.

Conversely, immune cell EVs can also promote inflammation. Wu et al. showed that EVs derived from adipose tissue can promote pro-inflammatory macrophage polarization. Likewise, Lamorte et al. proved that EVs from acute myeloid leukemia can alter the methylation profile of hematopoietic stem cells, promoting a pro-inflammatory state.

These varied mechanisms illustrate that EVs are not passive bystanders; they are active modulators of the immune microenvironment.

In acute inflammation, such as that seen in early infection or injury, EVs can help quickly mobilize immune cells and activate inflammatory pathways necessary to contain the insult. EVs derived from epithelial cells in infected tissues frequently carry danger signals that enhance immune surveillance and pathogen clearance. However, in chronic inflammation, the role of EVs becomes more nuanced. Diseases such as metabolic-associated fatty liver disease (MAFLD) are characterized by sustained production of EVs that perpetuate inflammatory signaling. These EVs often carry pro-inflammatory lipids, cytokines, and genetic material that reinforce immune cell recruitment and activation long after the initial trigger has passed. Wang et al. present an extensive review of progress and perspectives in EV-related research in MAFLD. Likewise, Garza et al. showed that exercise alters the profile of EVs in older adults, suggesting a shift in intercellular communications upon physical activity in a sex-dependent manner. Together, these studies highlight that rather than being simple markers of inflammation, EVs could actively shape the transition from acute to chronic inflammatory states, which underlies many pathological processes, as well as promote balanced immune responses in health.

Lastly, a rapidly expanding area of research concerns EVs derived not from human cells but from microorganisms. These microbial EVs can enter host tissues and influence immune responses, effectively acting as inter-kingdom messengers that link microbial behavior to host responses, which is summarized in an elegant review by Charpentier and Stanton. For example, Xin et al. and Glamočlija et al. showed that microbial-derived EVs could attenuate airway allergic responses in two different *in vivo* models. Likewise, da Silva et al. proved that EVs from bacteria could attenuate murine norovirus replication, suggesting that the impact of host-pathogen crosstalk via EVs affects a diverse range of pathogens and disruptions in this communication network have been implicated in inflammatory bowel disease, systemic autoimmunity, and even neuroinflammatory conditions (Wang et al.).

Overall, the work published in this Research Topic underscores the breadth of EV functions, origins, and therapeutic applications. It captures a transformative moment in immunological science. EVs are no longer peripheral curiosities; they are central agents of intercellular communication with profound implications for how inflammation is initiated, propagated, and resolved. From innate immune activation to chronic disease progression, from host-microbiota interactions to therapeutic design, EVs are rewriting the rulebook of inflammatory biology.

As we advance our understanding, EVs may not only illuminate the hidden language of cellular communication but may also become powerful tools in the diagnosis and management of inflammatory diseases—a frontier that holds immense promise for the future of medicine.

